# WW Domains of the Yes-Kinase-Associated-Protein (YAP) Transcriptional Regulator Behave as Independent Units with Different Binding Preferences for PPxY Motif-Containing Ligands

**DOI:** 10.1371/journal.pone.0113828

**Published:** 2015-01-21

**Authors:** Manuel Iglesias-Bexiga, Francisco Castillo, Eva S. Cobos, Tsutomu Oka, Marius Sudol, Irene Luque

**Affiliations:** 1 Department of Physical Chemistry and Institute of Biotechnology, Faculty of Sciences, University of Granada, 18071, Granada, Spain; 2 Weis Center for Research, Geisinger Clinic, M.C. 26–08, 100 North Academy Avenue, Danville, PA, 17822–2608, United States of America; Institute of Molecular and Cell Biology, SINGAPORE

## Abstract

YAP is a WW domain-containing effector of the Hippo tumor suppressor pathway, and the object of heightened interest as a potent oncogene and stemness factor. YAP has two major isoforms that differ in the number of WW domains they harbor. Elucidating the degree of co-operation between these WW domains is important for a full understanding of the molecular function of YAP. We present here a detailed biophysical study of the structural stability and binding properties of the two YAP WW domains aimed at investigating the relationship between both domains in terms of structural stability and partner recognition. We have carried out a calorimetric study of the structural stability of the two YAP WW domains, both isolated and in a tandem configuration, and their interaction with a set of functionally relevant ligands derived from PTCH1 and LATS kinases. We find that the two YAP WW domains behave as independent units with different binding preferences, suggesting that the presence of the second WW domain might contribute to modulate target recognition between the two YAP isoforms. Analysis of structural models and phage-display studies indicate that electrostatic interactions play a critical role in binding specificity. Together, these results are relevant to understand of YAP function and open the door to the design of highly specific ligands of interest to delineate the functional role of each WW domain in YAP signaling.

## Introduction

The Yes kinase-associated protein, YAP, is a potent oncogene and stemness factor [1, 2]. As a transcriptional co-activator, YAP elicits its oncogenic action *via* a two-prong strategy: it up-regulates genes that promote cell proliferation and also targets a set of genes that inhibit apoptosis [3]. YAP is a transforming gene that is amplified in several human cancers including breast, ovary, head and neck, and liver [4]. At present, the YAP gene and its products are studied intensively as YAP is one of the two main effectors of the newly delineated tumor suppressor pathway known as Hippo. The activation of the Hippo pathway by cell-to-cell contacts results in YAP phosphorylation on Serine 127 by Large Tumor Suppressor homolog (LATS) kinases. This modification anchors YAP in the cytoplasm via its interaction with the 14–3–3 protein, preventing YAP from nuclear localization and impairing transcriptional activity. The Hippo pathway was recently shown to crosstalk with a number of major signaling pathways including Notch, Wnt and TGF beta [1].

Cloning of the YAP gene resulted in the identification of a small modular protein domain, known as the WW domain, named after two conserved tryptophan residues spaced 20 to 22 residues apart within the sequence. WW domains are abundant and versatile protein-protein interaction modules that recognize proline-rich motifs [5] and adopt a common three-stranded antiparallel β-sheet fold [6, 7]. The side chain of the first conserved tryptophan lies on one side of the β-sheet in the hydrophobic core and is required for domain stability. The second tryptophan is located at one of the pockets at the binding site dedicated to proline recognition, the xP pocket, and common to most of WW domains. A second pocket in the binding site is responsible for binding specificity within WW domains subfamilies. In the case of type-1 WW domains, which preferentially recognize PPxY sequences, this specificity pocket is dedicated to the recognition of the Tyr residue in the consensus core-motif [8, 9].

**Figure 1:**
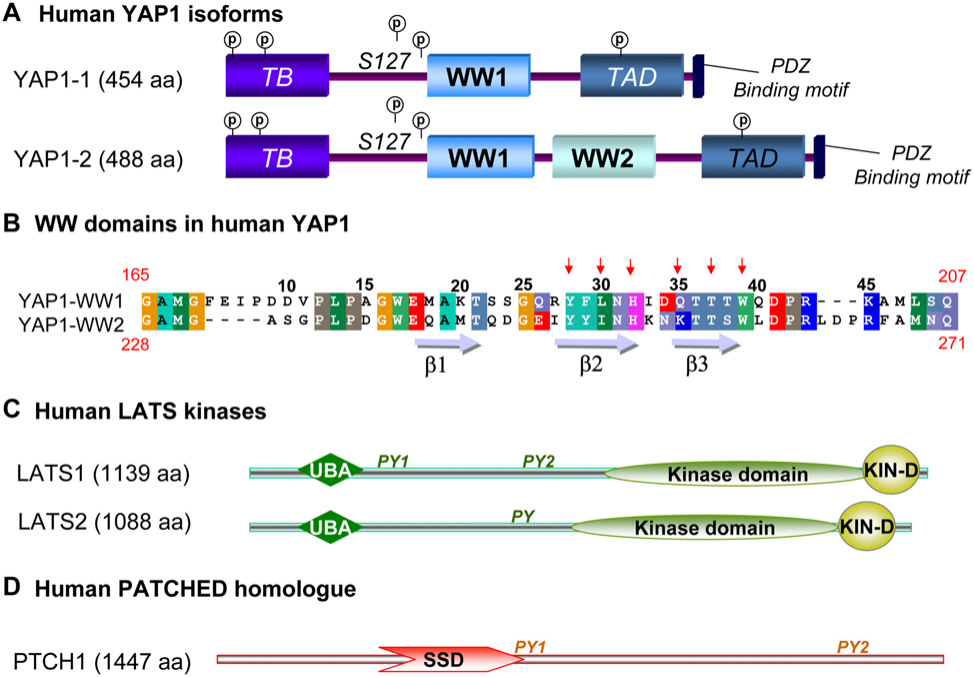
A) Modular organization of the two isoforms of human YAP transcriptional regulator. Both forms are comprised of one TEAD transcription factor-binding domain (TB), one (YAP-WW1) or two (YAP-WW1 and YAP-WW2) WW domains, one trans-activation domain (TAD) and one PDZ binding motif. The symbol P indicates the proposed serine phosphorylation sites. **B) Sequence alignment of the individual WW domains of human YAP1 and YAP2 isoforms**. Identical residues are shown on a black background and conservative changes on a gray background. Black arrows indicate those residues constituting the xP and xY pockets at the binding site. Red numbers indicate their position in the sequence of the full protein. **C) Modular organization of the human LATS1 and LATS2 kinases** containing an ubiquitin-associated domain (UBA), a C-terminal protein kinase domain (KIN-D) and one or two PY sequences. **D) Modular architecture of human PATCHED homologue 1** including a Sterol-sensing domain (SSD) and two PY sequences.

YAP is characterized by a well differentiated modular architecture (Fig. 1A), containing a transcriptional enhancer factor-binding domain (TB), a 14–3–3 binding-site, one or two type-1 WW domains, an SH3 binding motif, a transcriptional activation domain (TAD), a PDZ binding motif and several serine phosphorylation sites distributed throughout the sequence. WW domains are critical for the interaction of YAP with LATS kinases (Fig. 1C) and these domains also play a role in YAP ability to regulate transcription, cell transformation and tissue growth [10, 11]. There are two major isoforms of YAP that differ exclusively in the number of WW domains: YAP1 (also named Yap1–1gamma; Uniprot code: P46937–6), which contains only one WW domain and YAP2 (also known as Yap1–2 gamma; Uniprot code: P46937–1) containing two WW domains. YAP2, which is predominantly expressed in neural tissues, is considered the canonical sequence of YAP. Even though the precise signaling differences among YAP splicing variants remain to be elucidated, some differences in the behavior of the two isoforms have been reported. In this way, only YAP2 can interact with p73 [12] and AMOTL1 (Angiomotin-Like-1) [13]. Also, YAP2 has been described to be a more potent transcriptional co-activator of ErbB-4 than YAP1 [14].

Tandem repeats of modular domains are frequently found in signaling proteins, as a mechanism of optimization of cellular signal transduction. Several instances of WW domain tandems have been described, reporting different degrees of cooperativity between the constituting WW domains [15]. The elucidation of the degree and mechanism of co-operation between the two domains, the delineation of their binding specificity and the identification of proteins that associate with each of them is of relevance for a full understanding of YAP function as a regulator of the balance between proliferation and apoptosis within the Hippo signaling network. With this aim we have performed a detailed thermodynamic analysis of the structural stability and binding properties of the two WW domains of YAP, both as isolated domains and as domains in a tandem arrangement using calorimetric techniques and a set of functionally relevant ligands including peptide sequences derived from well-established functional partners of YAP, such as LATS kinases [12] and from Patched protein homolog 1 (PTCH1), a protein from the SHH pathway containing two PPxY motifs predicted as a cognate ligand of YAP WW domains (Fig. 1D). In order to probe PTCH1 as a ligand of YAP, functional studies in a cell culture model were performed.

Our work reveals that the WW domains of YAP behave as two independent units, both in terms of structural stability and ligand recognition, with no overt evidence of cooperativity between them. Binding studies show that the two WW domains exhibit different binding preferences, showing high selectivity for some ligands, such as the second PPxY motif in PTCH1. These results suggest that the presence of the second WW domain in YAP2 isoform modulates YAP function through partner recognition. Phage display studies and analysis of structural models of the different complexes suggest that electrostatic interactions play a key role in determining individual binding specificity for the WW1 and WW2 domains in human YAP.

## Materials and Methods

### Protein cloning, expression and purification

The actual boundaries of modular protein domains are not easy to define from their linear sequences (see Discussion in [16]). In the case of the WW domains, a demarcation of the functional length of the domain was facilitated by the naturally occurring splicing event that added one extra WW domain in the YAP2 isoform [17]. This second WW domain is 38 amino acids long and it is encoded, in the human YAP gene, by a single exon [5, 18]. In published reports, WW domains of various lengths have been used for structural and functional studies, ranging from 36 [19, 20, 21, 22] to 56 amino acids [17, 22]. Considering several studies that reported expression problems for shorter WW domain sequences [20, 23], we have expressed YAP WW domains as 46 amino acids long domains with the two conserved tryptophan residues centered in the sequence, as described in [6, 24, 25]. Accordingly, in the Uniprot P46937–1 entry the constructs used in this study correspond to residues 165–209 for the first WW domain of human YAP (YAP-WW1), residues 228–271 for the second WW domain (YAP-WW2), and residues 165–271 for tandem construct (YAP-WW1-WW2). The corresponding amino acid sequences for the two WW domains are shown in Fig. 1B).

The cDNAs for these constructs were synthesized by GENEART AG (Regensbug, Germany). All gene fragments were subcloned into the pETM-30 vector (Protein Expression and Purification Core Facility, EMBL, Heidelberg, Germany) for their expression as fusion proteins containing an N-terminal poly-histidine tag together with a Glutathione-S-Transferase (GST) tag and a Tobacco Etch Virus (TEV) protease cleavage site, for the removal of the affinity tags. Plasmid-encoding WW domain constructs were expressed in a BL21 (DE3) strain of *E. Coli* cells (Novagen). Cells were grown in Luria Bertani media at 37°C until OD_600nm_ ≈ 0.7. At this point, expression was induced with 0.15 mM IPTG for 5 hours at 37ºC. All constructs were purified by Ni-NTA affinity chromatography as previously described [26]. Protein-containing fractions were concentrated to 2 mg·mL^-1^ in 50 mM sodium phosphate 300 mM sodium chloride pH 8.0 buffer, frozen in liquid nitrogen and stored at -80º C. Under these conditions, protein samples were stable for several months.

YAP2 WW domain mutants (W-P to A-A) and PTCH1 PPxY mutants (PPxA) were generated as described before for YAP2 and LATS1 respectively [12].

### Peptide ligands

Synthetic peptide ligands were purchased from Peptide 2.0 Inc. (Chantilly, USA). All peptides were acetylated and amydated at their N and C termini, respectively. They were synthesized in the solid phase and their molecular mass was confirmed by mass spectrometry. Peptide purity was assessed by analytical HPLC as greater than 98%.

### Determination of protein and peptide concentration

Protein concentration was measured by absorbance at 280 nm using molecular weights of 5524 Da, 5410 Da, and 12529 Da and extinction coefficients of 12550 cm^-1^·M^-1^, 13960 cm^-1^·M^-1^ and 26400 cm^-1^·M^-1^, for YAP-WW1, YAP-WW2 and YAP-WW1-WW2, respectively. Their molecular mass was confirmed by mass spectrometry and their extinction coefficients were determined as described by Gill & von Hippel [27]. Peptide concentration was determined by absorbance at 278 nm using an extinction coefficient of 1450 M^−1^·cm^−1^ per tyrosine residue for ligands LATS1-a, PTCH1-a, and LATS1-b. For PTCH1-b, containing one tryptophan residue, an extinction coefficient of 6990 M^−1^·cm^−1^ at 280 nm was used.

### Differential Scanning Calorimetry (DSC)

The heat capacities of all samples were measured as a function of temperature using a high-precision differential scanning VP-DSC micro-calorimeter (Microcal Inc., Northampton, MA). Samples were prepared by extensive dialysis against a large volume of the appropriate buffers (20 mM sodium phosphate at pH 7.0, 20 mM sodium acetate at pH 5.0, 20 mM sodium acetate at pH 4.0 and 20 mM glycine at pH 3.0). All DSC experiments were performed at a scan rate of 1.5 K·min^-1^ using a protein concentration around 1.0 mg·mL^-1^. Protein samples and reference solutions were properly degassed according to manufacturer’s instructions and carefully loaded into the cells to avoid bubble formation. Thermal denaturation scans were recorded from 5 ºC to 110 ºC. Samples were cooled down inside the calorimeter and reheated to check the reversibility of the unfolding process at each experimental condition. The DSC thermograms were systematically corrected for the time-response of the calorimeter as well as for the instrumental baseline obtained with both calorimeter cells filled with the corresponding dialysis buffer. After normalization for protein concentration, the partial molar heat-capacity curves (*C*_p_) were calculated from the resulting thermograms, assuming a value of 0.73 mL·g^−1^ for the partial specific volume of all proteins.

The resulting DSC traces for isolated WW domains at different pH values were fitted individually and globally to a two-state (N⇄U) as described before [26, 28]. Nonetheless, in this case, the pH dependence of the unfolding heat capacity difference was explicitly included in the model (see definition in Supporting Information), according to the following equation:

$${\Delta \text{C}}_{\text{pN}-\text{U}}={\text{C}}_{\text{pU}}-{\text{C}}_{\text{pN}}+{\text{F}}_{\text{p}}\text{(pH)}\cdot {\Delta \text{C}}_{\text{p,prot}}$$ (equation 1)

, where ΔC_p,prot_ is the protonation heat capacity change, defined here as the difference in heat capacity at 100 ºC between the DSC traces at pH 7.0 and 3.0. ΔC_p,prot_ was considered as a fixed parameter in the global analysis. A value of 1.0 kJ·mol^-1^ was estimated from the experimental DSC traces for both individual domains (YAP-WW1 and YAP-WW2), which is in agreement with theoretical estimates considering the maximum contribution of all charged amino acid side chains in these domains [29]. F_p_ is the protonation fraction at each pH and was considered as an additional floating parameter for each pH condition. All DSC fittings and calculations were performed with Sigma Plot 2000 (Systat Software Inc., Chicago, USA).

### Isothermal Titration Calorimetry (ITC)

ITC experiments were carried out using a high-precision VP-ITC titration calorimeter (Microcal Inc., Northampton, Massachusetts). The WW domains were extensively dialyzed against 40 mM sodium phosphate buffer, pH 7.0, with the exception of those experiments using the LATS2 ligand, (containing a cysteine residue), for which the dialysis was performed in the presence of 10mM β-mercaptoethanol to reduce intermolecular di-sulphide bonds formed during ligand storage. Protein and peptide solutions, ranging from 20 to 40 μM for the proteins and from 0.8 to 2 mM for the ligands, were properly degassed to avoid bubble formation and equilibrated to 25°C prior to the titration experiment. In all cases, a profile of injection volumes from 3 to 20 μl was used to better define the titration curve. The heat evolved after each peptide injection was obtained from the integral of the calorimetric signal. The heat associated with the binding process was obtained as the difference between the heat of reaction and the corresponding heat of dilution, as obtained from independent titrations of the peptides into the buffer.

For the individual WW domains and those ligands with similar binding affinities for YAP-WW1 and YAP-WW2 domains (LATS1-a and LATS2), the resulting binding isotherms were analyzed by non-linear least-squares fittings to a model corresponding to a single set of identical sites, according to the equation:

$$\text{Q}=\frac{{\text{V}}_{0}\Delta \text{H}}{2{\text{K}}_{\text{a}}}\left[1+{\text{K}}_{\text{a}}\left[{\text{L}}_{\text{t}}\right]+\text{n}{\text{K}}_{\text{a}}\left[{\text{M}}_{\text{t}}\right]+\sqrt{{\left(1+{\text{K}}_{\text{a}}\left[{\text{L}}_{\text{t}}\right]+\text{n}{\text{K}}_{\text{a}}\left[{\text{M}}_{\text{t}}\right]\right)}^{2}-4\text{n}{\text{K}}_{\text{a}}^{2}\left[{\text{M}}_{\text{t}}\right]\left[{\text{L}}_{\text{t}}\right]}\right]$$ (equation 2)

, where Q is the net heat of binding, n is the number of binding sites, ΔH is the change in the enthalpy due to the binding process, K_a_ is the association constant, V_0_ is the active cell volume, and [M_t_] and [L_t_] are the total concentrations of macromolecule and the ligand, respectively.

For those ligands characterized by different binding affinities for the two WW domains (LATS1-b and PTCH1-a), analysis of the binding isotherms corresponding to the titration of the tandem construct were also analyzed according to a model of two sets of independent binding sites, n_1_ and n_2_. According to this model, the net heat of binding can be expressed as:

$$\text{Q}={\text{V}}_{0}\left[{\text{M}}_{\text{t}}\right]\left[\Delta {\text{H}}_{1}\frac{{\text{n}}_{1}{\text{K}}_{1}\left[{\text{L}}_{\text{t}}\right]}{1+{\text{K}}_{1}\left[{\text{L}}_{\text{t}}\right]}+\Delta {\text{H}}_{2}\frac{{\text{n}}_{2}{\text{K}}_{2}\left[{\text{L}}_{\text{t}}\right]}{1+{\text{K}}_{2}\left[{\text{L}}_{\text{t}}\right]}\right]$$ (equation 3)

, where Q is the net heat of binding, n_1_ and n_2_ are the number of sites of each class, ΔH_1_ and ΔH_2_ are the change in the enthalpy due to the binding process for each site, K_1_ and K_2_ are the association constants for each site, V_0_ is the active cell volume, and [M_t_] and [L_t_] are the total concentrations of macromolecule and the ligand, respectively.

Both models were implemented in Origin 7.0 software (OriginLab Corporation, Northampton, MA). For the least-squares fit using the one set of sites model, the number of binding sites, the association constant, and the binding enthalpy were considered as floating parameters, while in the analysis with the two set of sites model, only the two association constants and the two binding enthalpies were left to float. All experiments were performed at least twice. Typically, the variability of the experimental values was estimated to be about 1% in the number of binding sites, 5% in the binding enthalpy and 10% in the binding affinity.

### Cell Culture and Transfections

HEK293 cells were cultured in Dulbecco’s modified Eagle’s medium (DMEM) supplemented with 10% fetal calf serum. The semi-confluent cells we transiently transfected with DNA constructs using Lipofectamine (Invitrogen), as specified by the manufacturer.

### Plasmids

YAP cDNA, wild type (WT) in pcDNA4/His-Max vector and p73 HA tagged expression vector were as described previously [12]. Human PTCH1 cDNA (full length, 2172 bp) was sub-cloned into pFLAG CMV2 vector into KpnI and XhoI sites. The FLAG-tagged PTCH1 was then sub-cloned into pcDNA6/TR vector from Invitrogen. This vector encodes TET repressor and was used to establish stable, inducible HEK293 cell line. The same method as the one used for the establishment of YAP inducible HEK293 cells was used for the FLAG-tagged PTCH1 inducible cells [12].

### Cell counting assay

HEK293 cultivated in 1% FBS serum were induced by Tetracycline to express Flag-PTCH1 protein as described previously for YAP overexpression in HEK 293 cell [12]. Cells were trypsinized and immediately counted using Beckman Coulter cell counter.

### Immunoprecipitation assay

Lysates of HEK 293 cells, the RIPA buffer used to lyse cells and FLAG M2 antibody from Sigma company and YAP polyclonal rabbit antibody used in our assays, plus all other steps in the analysis of immuno-precipitates on polyacrylamide gels and on western blots were exactly as described previously [12].

### Homology modeling of YAP complexes and calculation of electrostatic potential

Homology modeling of isolated WW domains and their complexes with the different peptides used in this study (LATS1-a, LATS1-b, LATS2, PTCH1-a and PTCH1-b) was performed using the Discovery Studio Suite (Accelrys Inc., San Diego, USA). The homologue search and sequence alignment was performed against locally installed databases using BLAST and PSI-BLAST. Multiple sequence alignments were calculated using sequence and structure information of the protein family. A well-defined NMR structure of the complex between a YAP-WW1 mutant and a PPPY containing peptide [6] (PDB code 1JMQ) was chosen as a template for the modeling of YAP-WW1 and YAP-WW2 complexes with the different ligands. The final 3D models were generated using the program MODELLER [30] and the lowest-energy model was selected in each case. For each WW domain-peptide complex, the ligand was built by mutation of the peptide in the 1JMQ template and was properly oriented by superposition with the template using the Discovery Studio Suite. The protonation state of ionizable residues in each model was determined using PDB2PQR software [31]. All models underwent an energy minimization cycle in vacuum using the AMBER ff10 force field [32]. The quality of the models was evaluated using PROCHECK [33]. The hydrogen bond interactions in the protein-ligand interfaces were evaluated with the MM-ISMSA software [34] considering that the distance between the hydrogen and acceptor atoms is within 1.5 Å and 2.4 Å, the angle between donor, hydrogen, and acceptor atoms varies within 130º and 165º; and the angle between the hydrogen, acceptor, and the atom bound to the acceptor atom diverges between 115º and 145º. The electrostatic potential of the protein and ligands was calculated from the model structures using the DelPhi algorithm [35], as implemented in the Discovery Studio Suite. Before running DelPhi, atom charges were assigned according to the CHARMm force-field parameters [36].

### Phage-display study of WW domain binding preferences

The analysis of the binding preferences of the WW domains was performed by phage-display as previously described [37]. Briefly, an N-term library in PIII was generated using a template derived from the pS2202d phagemid, containing the Erbin-PDZ domain kindly provided by Dr. Sachdev Sidhu (University of Toronto). The Erbin-PDZ sequence was removed by the introduction of four stop codons between the secretion signal and the linker required for the correct display of the library variants in the phage. A randomized sequence corresponding to a x_5_(L/P)PxYx_5_ peptide library containing the L/PPxY core motif for type 1 WW domains was subsequently introduced into the modified phagemid. The resulting library was then introduced into the SS320 strain by electroporation. The subsequent phage propagation led to 10^11^ library diversity, which was isolated at 5 x 10^13^ phages/ml in PBS-Tween (0.05%) buffer. The resulting library was then screened against immobilized GST-tagged YAP-WW2 domain (GST-YAP-WW2) in PBS, carrying out seven rounds of an iterative selection process. Single clones were chosen from round 3 to round 7 and tested for specific binding to the GST-YAP-WW2 by phage ELISA in a Tecan Infinite M200 instrument [37, 38]. In parallel, additional binding tests were performed in the presence of p53BP2 peptide (EYPPYPPPPYPSG) [39], which was used as a competing agent for the selection for high-affinity sequences. Additionally, in order to evaluate the level of specificity of the selected sequences for the two WW domains of YAP, the single clones derived from the GST-YAP-WW2 selection process were also tested for their ability to displace p53BP2 from the YAP-WW1 domain. The DNA from phages showing binding in the ELISA assay were used as PCR templates to amplify the peptide-encoding regions, which were then sequenced and analyzed.

## Results

### YAP WW domains are marginally stable and behave as independent folding units connected by an unstructured linker

The temperature dependence of the partial heat capacity of the isolated YAP-WW1, YAP-WW2 domains and the YAP-WW1-WW2 tandem was measured by Differential Scanning Calorimetry (DSC) at different pH values, ranging from pH 7.0 to pH 3.0. The reversibility of the unfolding transitions was checked and confirmed to be over 80% in all cases. No scan rate or concentration effects were observed for any of the constructs, indicating that they behave as monomeric proteins that unfold in equilibrium under all studied conditions.

**Figure 2:**
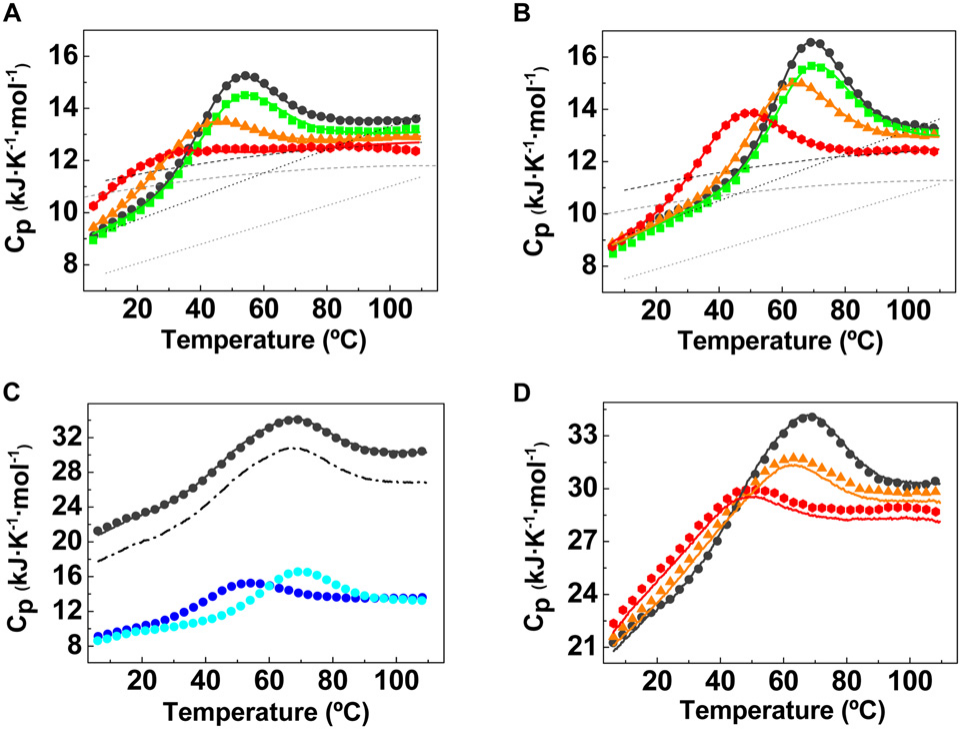
Differential Scanning Calorimetry thermal denaturation profiles of YAP1-WW1 (A) and YAP1-WW2 (B) domains. Symbols correspond to experimental data for the temperature dependency of the partial molar heat capacity at different pH values (black circles for pH 7.0, green squares for pH 5.0, orange triangles for pH 4.0 and red rhombi for pH 3.0). Solid lines correspond to the global fitting to the two-states model. The heat capacities functions for the folded and unfolded states [C_p,N_(T) and C_p,U_(T)] resulting from the analysis are shown as short dashed lines. The dotted lines show the C_p,N_(T) function calculated according to the molecular weight [28] and the C_p,U_(T) function estimated from the contributions of the amino acid composition [41]. **C) DSC profile for the YAP1-WW1-WW2 tandem**. Shown are the DSC profiles for YAP-WW1 (dark blue circles), for YAP-WW2 (light blue circles) and YAP-WW1-WW2 tandem (black circles) at pH 7.0. The dashed line corresponds to the addition of the heat capacity profiles of the individual WW domains and the continuous black line represents the addition of the DSC experiments of the individual WW domains plus the contribution to the heat capacity for the linker sequence in the tandem [41]. **D) DSC profile for the YAP1-WW1-WW2 tandem at different pH values.** Symbols correspond to experimental data for the temperature dependency of the partial molar heat capacity of the tandem at different pH values (black circles for pH 7.0, orange triangles for pH 4.0 and red rhombi for pH 3.0). The continuous lines correspond to the curves resulting from the addition of the heat capacity profiles for the individual WW domains and linker sequences under each condition.

The two isolated domains were characterized by very broad calorimetric transitions (Fig. 2A and B). To minimize the errors in the determination of the unfolding thermodynamic parameters the denaturation curves for each WW domain at different pH values were globally analyzed by non-linear least squares fitting to a two-state model [26] modified to incorporate ionization effects to the unfolded heat capacity to account for the progressive increment in C_p,U_ with pH, which in these small and broad transitions become relevant (S1 Table). The results of this analysis are summarized in S2 Table. At pH values between 4.0 and 7.0, both YAP-WW1 and YAP-WW2 are fully folded below 20 ºC. YAP-WW2 shows a slightly higher stability than YAP-WW1 (S1 Fig.). Even though the two-state model adequately describes the experimental data (R and R^2^ values of 0.99), the values of the thermodynamic parameters resulting from the two-states analysis are not within the normal range expected for a standard two-state protein (S2 Table). This suggests that, in as previously discussed for other WW domains [26, 40, 41], the isolated WW domains of YAP are highly flexible and marginally stable units in the limits of what can be considered cooperative folding.

The YAP-WW1-WW2 tandem construct (Figs. 2C and D) presents a single and broad transition that cannot be described by a two-state model. As illustrated in Fig. 2C and 2D, at all pH values the DSC profile of the tandem can be perfectly reproduced by addition of the DSC curves of the YAP-WW1 and YAP-WW2 domains plus the contribution of the heat capacity of a disordered linker [24], calculated as the sum of the tabulated Cp contributions of the different amino acids in the sequence [42]. This indicates that the two WW domains in tandem unfold independently, without any significant cooperative interactions between them or with the connecting linker. In other words, the presence of the second WW domain in YAP2 does not lead to the stabilization of the first WW domain nor to a conformational reorganization of the tandem. We hypothesize, thus, that the presence of the second WW domain in the YAP2 isoform results in the modulation of the target recognition properties of YAP by inducing changes in binding affinity and/or specificity towards their cellular partners.

### Selection of ligands for YAP WW domains

To investigate this hypothesis, a thermodynamic analysis of the interactions of the isolated WW domains and the YAP-WW1-WW2 tandem was performed using a set of PPxY containing ligands. LATS kinases play a key role in YAP functional regulation through Ser127 phosphorylation, and are well established as partners of YAP WW domains [12]. Accordingly, three different peptide ligands corresponding to the PPxY containing sequences in LATS1 and LATS2 were selected for the binding studies.

In order to widen the study, additional putative ligands of YAP WW domains were considered. Taking into account that YAP has been recently reported as a regulator of the Sonic Hedgehog Pathway [43, 44], we investigated if any members of this pathway contain PPxY motifs which could serve as targets for WW binding. Interestingly, we found that PATCHED1 (PTCH1) contained two PPxY motifs including one with four consecutive prolines (RYSPPPPYSSHS), which usually forms a core for high affinity peptide ligands to YAP WW domains [39].

**Figure 3:**
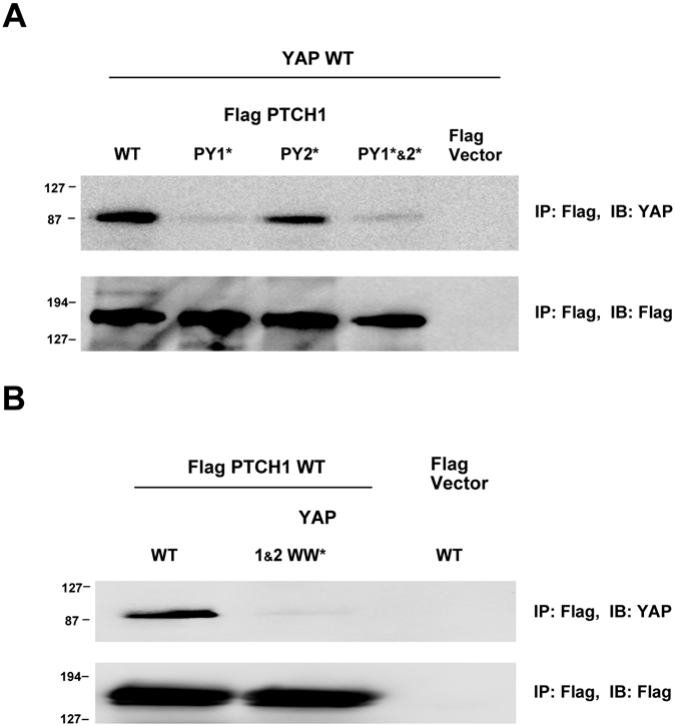
YAP interacts with PTCH1 Through its WW Domains. **A)** Intact PPxY motifs in PTCH1 are required for binding to YAP. Two PPxY sequences in PTCH1 were mutated to PPxA. Wild type (WT) or single (PY1* or PY2*) or double (PY1*&2*) mutants of PTCH1 in Flag-tag vector were transiently co-transfected with YAP2 (in pcDNA4/HisMax) into HEK293 cells. Cell lysates were immunoprecipitated with Flag antibodies, resolved on SDS-PAGE and immunoblotted with YAP antibody or Flag antibody. **B)** Intact WW domains in YAP are required for binding to PTCH1. Two WW domains in YAP were mutated to render them inactive in terms of ligand binding. Wild type (WT) or double (1&2 WW*) mutant of YAP2 in pcDNA4/HisMax vector were co-transfected with Flag-PTCH1 or Flag vector alone. Cells were lysed and analyzed as in A.

The interaction between PTCH1 and YAP was examined using PTCH1 and YAP mutants in a cell culture model. PTCH1 mutants in which the signature tyrosine residue (Y) in each PPxY motif was substituted with alanine were generated. Such mutation has been shown to abrogate binding of PPxY-containing ligands to WW domains [14, 22]. The resulting PTCH1 variants (the wild type protein, the single mutants labeled PY1* and PY2* and the double mutant labeled PY1*&2*) were fused to FLAG tags and transiently co-expressed with YAP in HEK293 cells, followed by immunoprecipitation and immunoblotting (Fig. 3A). In this experimental setting PTCH1 WT bound strongly to YAP (Fig. 3A, upper panel). Binding became weaker when the first PPxY motif was mutated (PY1* or PY1*&2*), while no significant effect was observed for the mutation of the second PPxY motif (PY2*). These data suggest that, in fact, PTCH1 and YAP interact and that the first PPxY motif in PTCH1 plays a critical role in the formation of the complex.

In order to test whether the PTCH1-YAP interaction is actually mediated by the YAP WW domains, two highly conserved amino acids in each of the two WW domains (i.e. the second signature W and the conserved, carboxy-terminal P) were mutated to A. The WQDP sequence of amino acids 199–202 in the first WW domain of YAP was changed to AQDA, while the WLDP sequence of amino acids 258–261 in the second WW domain of YAP was changed to ALDA. Such substitutions in WW domains render the mutated domains inactive in terms of ligand binding [14]. The double mutant was labeled as 1&2 WW*. This mutant and the YAP WT were co-transfected with Flag-PTCH1 into HEK293 cells, followed by immunoprecipitation and immunoblotting (Fig. 3B). Relatively strong binding was detected in the case of YAP WT. However, this binding was barely detectable when both WW domains of YAP were mutated. Together, the results suggest that the binding between YAP and PTCH1 is mediated by the WW domains of YAP (Fig. 3A and B). Experiments with a cell-apoptotic model also support the YAP-PTCH1 interaction (S3 Fig.), revealing that, in the presence of PTCH1, the effect of YAP expression in the cell growth is reduced and YAP also failed to stabilize p73, in agreement with the observed effects for the interactions with other YAP Partners [12].

Even though the cellular studies were performed with overexpressed proteins and do not provide conclusive evidence of the physiological relevance of this interaction, they provide validation to the idea that, at least in this setup, the PTCH1 PPxY sequences can interact with YAP WW domains. Consequently, we decided to incorporate the PTCH1 sequences to our binding studies. In summary, a set of five different peptide ligands containing 12 amino acids with the PPxY motif centered within the sequences corresponding to the PPxY motifs in LATS1, LATS2 and PTCH1, were selected for the binding studies (see Table 1 for sequences).

**Table 1:** Peptide ligands derived from YAP functional targets.

	**Ligand**	^1^Sequences
**Sonic Hedgehog Pathway**		
**PTCH1** *“Protein Patched Homolog 1”* G.lycosylated membrane receptor	**PTCH1-a**	^572^R_-7_Y_-6_S_-5_P_-4_ **P_-3_P_-2_P_-1_Y_0_**S_1_S_2_H_3_S_4_^583^
**PTCH1** *“Protein Patched Homolog 1”* Glycosylated membran e receptor	**PTCH1-b**	^1243^E_-7_G_-6_L_-5_W_-4_ **P_-3_P_-2_P_-1_Y_0_**R_1_P_2_R_3_R_4_^1254^
**Hippo pathway**		
**LATS1** *“Large Tumor Suppressor Homolog 1”* Serine/threonine kinase	**LATS1-a**	^369^N_-7_R_-6_Q_-5_P_-4_ **P_-3_P_-2_P_-1_Y_0_**P_1_L_2_T_3_A_4_^380^
**LATS1** *“Large Tumor Suppressor Homolog 1”* Serine/threonine kinase	**LATS1-b**	^552^Y_-7_Q_-6_G_-5_P_-4_ **P_-3_P_-2_P_-1_Y_0_**P_1_K_1_H_1_L^563^
**LATS2** *“Large Tumor Suppressor Homolog 2”*Serine/threonine kinase	**LATS2**	^511^R_-7_R_-6_C_-5_P_-4_ **P_-3_P_-2_P_-1_Y_0_**P_1_K_2_H_3_L_4_^522^

^1^Numbers indicate the position of each ligand in the context of the full-length protein sequence.

### The two WW domains of YAP2 act as independent docking sites with different binding properties

**Figure 4:**
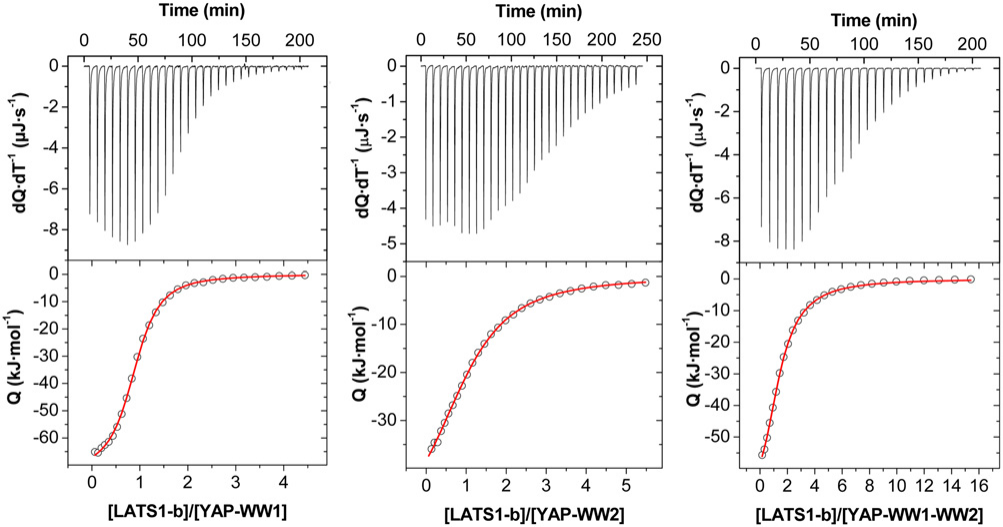
Isothermal titration calorimetry experiments for YAP1-WW1, YAP1-WW2 and YAP-WW1-WW2 tandem with the LATS1-b ligand at 25ºC in 20 mM sodium phosphate pH 7.0. Upper panels show the heat effects associated with the injection of LATS1-b ligand into the calorimetric cell containing the protein. Lower panels show the corresponding binding isotherm ligand normalized by protein concentration corrected for the heat of dilution. Solid lines correspond to the best fit of data to a one-site model (equation 2) in the experiments with individual WW domains and to a two sets of independent binding sites model (equation 3) in the experiment with the tandem (right panel).

The binding energetics of all peptide ligands to each YAP WW domain and to the tandem construct were measured by Isothermal Titration Calorimetry (ITC). The upper panels in Fig. 4 show, as an example, the calorimetric titrations of the LATS1-b ligand (YQGPPPPYPKHL) with each construct. The corresponding binding isotherms are shown in the lower panels. The results of the thermodynamic analysis for the isolated domains are summarized in Table 2 and illustrated in S2 Fig. The ITC experiments confirmed that all five PPxY-containing ligands bind to at least one of the WW domains of human YAP with K_d_ values in the low μM range. It is interesting to note that each of the LATS kinases contain at least one ligand with dissociation constants close to or below 10 μM for YAP-WW1. This is the case for the only PPxY-containing sequence in LATS2 and for one of the two PPxY-containing peptides derived from LATS1 and PTCH1 (LATS1-b and PTCH1-b), with K_d_ values of 7.1, 3.7 and 13.2 μM, respectively. These values are close to those previously reported for the p53BP2 ligand, characterized by the highest binding affinity measured to date for YAP WW domains (K_d_ = 1.8 μM for YAP-WW1 and 12 μM for YAP-WW2) [39]. It is interesting to note that, *in vitro*, the two isolated PPxY-containing sequences in this protein can interact with YAP-WW1 with dissociations constants in the low micromolar range, being the binding affinity of the PTCH1-b ligands slightly higher than the PTCH1-a. Nonetheless, in the cellular assays, mutation of the second PPxY motif (PTCH1-b) in the context of the full-length proteins had a much smaller effect on PTCH1-YAP binding. For all ligands studied, the K_d_ values for the tandem construct do not differ significantly from those obtained for the individual domains, suggesting that the WW domains in YAP behave as two independent binding sites for these peptide ligands.

**Table 2:** Binding thermodynamics of peptide ligands to YAP WW domains.

**Protein**	**Ligand**	**n**	**K_d_ (μM)**	**ΔG_ap_ (kJ·mol^-1^)**	**ΔH_ap_ (kJ·mol^-1^)**	**-T·ΔS_ap_ (kJ·mol^-1^)**
**YAP-WW1**	**PTCH1-a** RYSPPPPYSSHS	0.95	27.6	-26.1	-65.0	38.9
	**PTCH1-b** EGLWPPPYRPRR	1.09	13.2	-27.9	-75.2	47.3
	**LATS1-a** NRQPPPPYPLTA	0.96	21.5	-26.7	-67.0	40.3
	**LATS1-b** YQGPPPPYPKHL	0.91	3.7	-31.0	-69.0	38.0
	**LATS2** RRCPPPPYPKHL	1.05	7.1	-28.8	-78.5	49.7
**YAP-WW2**	**PTCH1-a** RYSPPPPYSSHS	0.91	45.8	-24.8	-65.9	41.1
	**PTCH1-b** EGLWPPPYRPRR	n.b.	—	—	—	—
	**LATS1-a** NRQPPPPYPLTA	1.09	27.0	-26.1	-76.2	50.1
	**LATS1-b** YQGPPPPYPKHL	0.94	33.9	-25.6	-70.2	44.6
	**LATS2** RRCPPPPYPKHL	1.08	8.2	-29.1	-57.8	28.7
**YAP-WW1-WW2**	**PTCH1-a^2^** RYSPPPPYSSHS	1^3^	24.7	-26.4	-52.9	26.5
		1^3^	47.4	-24.7	-75.2	50.5
	**PTCH1-b^1^** EGLWPPPYRPRR	1.23	16.9	-27.3	-80.2	52.9
	**LATS1-a^1^** NRQPPPPYPLTA	2.04	33.4	-25.6	-72.0	46.4
	**LATS1-b^2^** YQGPPPPYPKHL	1^3^	4.8	-30.4	-70.8	40.4
		1^3^	31.9	-25.7	-51.8	26.1
	**LATS2^1^** RRCPPPPYPKHL	2.02	9.2	-28.8	-68.8	40.0

The variability in the experimental values was estimated to be about 1% for the number of sites, 5% for the binding enthalpy and 10% for the dissociation constant. n.b.: no binding detected.

^1^Data obtained using a binding model for one set of sites with n = 2.

^2^Data obtained using a binding model for two different sets of sites with n = 1 for each set of sites. (see methods for details).

^3^Number of binding sites fixed to 1 in fitting procedure.

In addition to binding affinity, ITC provides relevant information about the enthalpic and entropic components to the Gibbs energy of binding, which report on the type and magnitude of the forces driving the binding affinity [45], providing, thus, a valuable insight into the nature of the interactions. Binding of the WW domains of YAP with the five peptide ligands is driven by favorable enthalpy contributions, ranging from -53 to-80 kJ·mol^-1^, partially compensated for by unfavorable entropic contributions. This energetic signature is similar to that reported for other WW domain-mediated interactions [19, 46, 47] and for other proline-rich recognition domains such as SH3 [48, 49, 50] or UEV domains [51]. In the case of SH3 domains, this thermodynamic pattern has been associated to the interplay of several factors, including the redistribution of the native state conformational ensemble upon ligand binding [50, 52] and the presence of interfacial water molecules [53].

Interestingly, the analysis of the binding energetics of the five chosen ligands of YAP WW domains indicates that, in spite of the high sequence similarity between the binding sites of the two domains, they behave differently with respect to ligand recognition. Some YAP ligands, such as LATS1b, bind more tightly to YAP-WW1 than to YAP-WW2 (See Table 2 and S2 Fig.). These data are in agreement with co-immunoprecipitation assays of LATS and YAP using mutants of individual WW domains rendered inactive in terms of ligand binding [12]. These assays, which involved full-length proteins, also showed a more prevalent role of YAP-WW1 than YAP-WW2 domain in the formation of the YAP - LATS complex. This selectivity between the two WW domains is particularly evident for the PTCH1-b ligand, for which no interaction was detected with YAP-WW2.

Moreover, even for those ligands, such as PTCH1-a, LATS2 or LATS1-a, which do not seem to distinguish between the two WW domains of YAP, significant differences, up to 20 kJ·mol^-1^, are observed in their enthalpic and entropic contributions to the binding affinity. Even though these contributions are not translated into changes in binding affinity due to enthalpy/entropy compensation effects [54], they indicate that the balance of intermolecular forces driving the binding of these ligands is different for each WW domain. In sum, our biophysical data suggest that YAP-WW1 and YAP-WW2 are not equivalent modules with respect to ligand recognition.

### Electrostatic interactions play a key role in determining binding specificity between the two WW domains in human YAP

**Figure 5:**
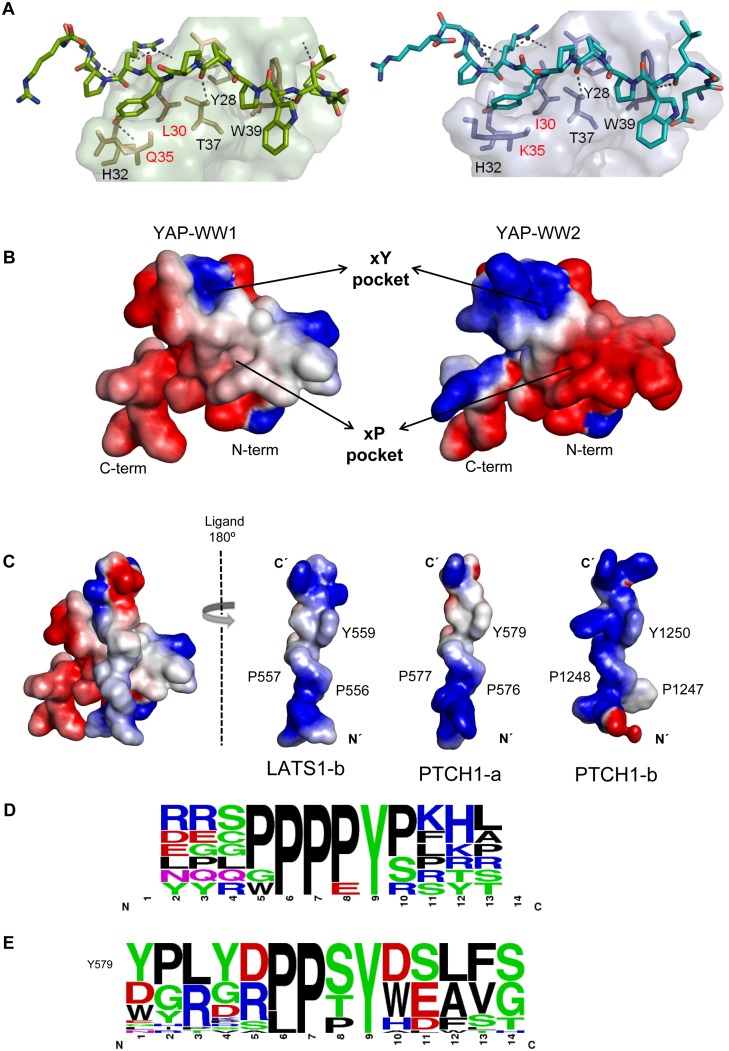
A) Cartoon representation of the YAP-WW1 (left) and-WW2 (right) domains in complex with PTCH1-b. YAP-WW1 and—WW2 domains are shown as green and blue surfaces respectively. Peptide ligands and protein residues defining the canonical xP and xY pockets at the binding sites are shown as sticks. Non-conserved residues at the binding site are labelled in red. Hydrogen bonds interactions are shown as discontinuous black lines. **B) Surface representation of the electrostatic potential calculated for the free YAP-WW1 and YAP-WW2 domains**. Regions of strong negative potential are shown in red and positively charged regions are shown in blue. **C) Surface representation of the electrostatic potential calculated for the YAP-WW1 domain/LATS1-b complex and free LATS1-a, PTCH1-a and PTCH1-b ligands**. Regions of strong negative potential are shown in red and positively charged regions are shown in blue. **D) Frequency of occurrence of amino acids in natural ligands binding preferentially to YAP1-WW1.** Web-logo [67] showing the frequency of occurrence of the different amino acids for a set of peptide ligands corresponding to natural proteins binding preferentially to YAP-WW1. **E) Frequency of occurrence of amino acids in phage-display selected ligands binding preferentially to YAP1-WW2.** Web-logo showing the frequency of occurrence of the different amino acids for the set of charged sequences selected by YAP-WW2 in phage display experiments, excluding the neutral, proline-rich sequence AGRPPPPPYPGPPL.

The most characteristic residues at the binding site of class I WW domains are conserved in both, YAP-WW1 and YAP-WW2 (see Fig. 1B), with the exception of Leu30 and Gln35 in YAP-WW1 that are substituted by Ile and Lys respectively in YAP-WW2. Accordingly, as illustrated in Fig. 5A and S4 Fig., the modeled structures for YAP-WW1 and-WW2 domains bound to the different peptide ligands are very similar and reproduce the main binding features characteristic of class I WW complexes. Hydrogen bond interactions and their proton-donor and acceptor distances for all complexes are summarized in S3 Table. All modeled complexes share a highly conserved pattern of hydrogen bond interactions within the canonical binding site, differing only with respect to the interactions established with polar and positively charged residues at the N-terminal regions of the ligands.

Electrostatic potential calculations carried out with the modeled structures for YAP-WW1 and YAP-WW2 (Fig. 5B), show that the Q35K and E25Q substitutions at the xY pocket and β2 strand respectively have a significant impact on the electrostatic potential of the two domains in the binding site area, leading to a marked polarization in the binding site of YAP-WW2, with a region of strongly positive electrostatic potential located over the ‘xY pocket’ and the loop connecting the β2 and β3 strands (including lysine 35) and a region of markedly negative potential corresponding to the ‘xP pocket’. However, this polarization is not observed in YAP-WW1, suggesting a relevant role of electrostatic interactions in the determination of binding specificity between these two domains.

In fact, analysis of the ligand sequences known to interact preferentially with YAP-WW1, including the LATS and PTCH1 peptides used in this study, and the recently described sequences from SMAD7 (ELESPPPPYSRYPM) and AMOT p130 (MRYQHPPEYGAARP) [55, 13], shows a clear preference for positively charged residues C-terminal to the PPxY core motif (Fig. 5D). These sequences present a region of positive potential, resulting in repulsive interactions with the ‘xY pocket’ at the YAP-WW2 binding site (See Fig. 5C). The PTCH1-b ligand constitutes an extreme situation, with a stretch of three positively charged arginines that would completely preclude interaction with YAP-WW2. Moreover, considering that electrostatic interactions contribute mostly to the binding entropy, the idea that electrostatic interactions play a prevalent role in determining binding specificity between these domains is in agreement with the fact that the differences in binding affinity found in this study between the two WW domains are of entropic origin (see Table 2).

In order to further investigate this idea, the binding preferences of YAP-WW2 were studied by phage display. From these experiments, a set of 42 peptides, with the ability to displace the p53BP2 peptide ligand (EYPPYPPPPYPSG) from the YAP-WW2 domain, were identified. These sequences are, thus, “high affinity” peptides expected to bind YAP-WW2, with dissociation constants below 12 μM for this domain. Two types of sequences were obtained. The first was a neutral proline-rich sequence, AGRPPPPPYPGPPL, which was selected a total of 14 times in the set of 42 peptides. This sequence is reminiscent of the p53BP2 ligand, although it includes an arginine at position 3, which is frequent in YAP ligands (Fig. 5D). As observed for the p53BP2 peptide ligand (unpublished data), ELISA binding experiments showed that this proline-rich peptide seems to interact similarly with the two WW domains of YAP. The second type of sequences obtained included peptides characterized by a relatively variable N-terminal region and, most interestingly, by the invariant presence of negatively charged residues C-terminal to the PPxY core motif. These sequences, shown in Fig. 5E, are markedly different from those derived from natural ligands binding preferentially to YAP-WW1 (Fig. 5D). From the ELISA assay results, contrary to the above-mentioned AGRPPPPPYPGPPL sequence, the second set of sequences seems to be highly selective for YAP-WW2, not being able to displace the p53BP2 ligand from the YAP-WW1 domain. These results indicate that the second set of sequences have a reduced binding affinity for YAP-WW1, in comparison to the YAP-WW2 domain. Interestingly, this is in agreement with the fact that p73 protein, which contains the sequence SHCTPPPPYHADPS, has been described to interact exclusively with the YAP2 isoform [12].

## Discussion

YAP, as one of the main effectors in the Hippo tumor suppressor pathway, is currently the subject of great interest and intense study. Elucidating how YAP regulates signaling in the context of this pathway is key to understand cell proliferation, differentiation and apoptosis. Furthermore, as information accumulates for Hippo and other cancer-related signaling cascades, the need to integrate this knowledge, elucidating the interplay between different pathways grows even more apparent.

The Hippo pathway has been recently reported to cross-talk with other signaling cascades, including TGFB, Wnt [56] and Notch [57, 58], controlling proliferation in different types of cells and acting as a central node integrating signals originated in other pathways (see [59] for a review). With respect to the interaction between Hippo and Sonic Hedgehog pathways, recent studies suggest that SHH signaling acts downstream of YAP in regulation of neuronal differentiation [44], being YAP either amplified or up-regulated in human SHH-associated medulloblastomas [43]. The results presented here suggest that PTCH1 might function as a cognate ligand of YAP, providing a first hint for a molecular link between these two pathways. Clearly, a more detailed analysis performed under physiological conditions and out of the scope of this article is needed to fully establish the physiological relevance of this potential cross-talk via PTCH1 and YAP that, if confirmed, could help identify new signaling events that regulate Hippo-YAP pathway.

In any case, we have shown that PTCH1 is able to interact with YAP in a cellular context and that this interaction is mediated by YAP WW domains, showing a marked preference for the WW1 domain. *In vitro*, the two isolated PPxY-containing sequences in PTCH1 can interact with YAP-WW1 with dissociation constants in the low micromolar range, which can be considered as a tight interaction in view of the range of binding affinities typically found for WW domains. Nonetheless, according to the results of the *in vivo* assays, in the context of full-length proteins the PTCH1-YAP interaction seems to be mostly driven by binding of the first PPxY motif in PTCH1 to the WW1 domain in YAP. Even though it is feasible that the second PTCH1-PPxY motif could act as a docking site for different signaling proteins in higher-level assemblies, the actual role of this PTCH1 sequence and whether it is implicated at all in the Hippo-SHH interplay remains to be elucidated. In any case, the discrepancies between *in vitro* and *in vivo* results highlight the importance of contextual effects in protein-protein interactions mediated by modular domains [60, 61] and the need to be cautious in the interpretation of *in vitro* results obtained with isolated peptides and domains in terms of cellular events.

With all these caveats, the thermodynamic study presented here provides, nonetheless, very valuable information in terms of the interplay between the two WW domains in YAP and their binding properties. Differential scanning calorimetry studies reveal that, both isolated and in a tandem disposition, these domains show marginal stability, with thermodynamic properties typical of downhill proteins, characterized by a poor cooperativity and, thus, high conformational flexibility [25]. This thermodynamic scenario set the best framework for a high plasticity in ligand recognition, in agreement with the functional diversity reported for these YAP WW domains [47, 55, 62]. Furthermore, no evidence of functional cooperativity between the two YAP WW domains was found, either in terms of structural stability, or with respect to the recognition of peptide ligands. This indicates that, in every aspect, these two domains function as independent docking sites. This independent behavior contrast with previous studies reporting on tandems of WW domains that function as a single unit connected by a structured linker, WW domains chaperoning each other by increasing their stability and physiological function, etc. [13, 63, 64]. According to the evidence presented here, the WW domains of YAP1 behave as two independent, fully folded, although marginally stable units, connected by an unstructured linker.

Most interestingly, the analysis of the binding energetics of the selected set of peptide ligands revealed that, in spite of the high similarity within the binding site region, the two WW domains in YAP interact differently with their ligands, as hinted by previous studies reporting modest differences (up to 5.3 kJ·mol^-1^) in binding affinity with other ligands [6, 22, 47, 55]. Nonetheless, to our knowledge, this is the first report of a high selectivity between the two WW domains in YAP. This selectivity becomes more relevant considering that differences in partner recognition have been reported for the two YAP isoforms (only YAP2 can interact with p73 [12] and AMOTL1 (Angiomotin-Like-1) [65]. Moreover, it has been proposed that YAP may affect different signaling pathways in a cell-specific manner, interacting with different transcription factors in different cell types, and functioning differently depending on its binding partners [44]. In this context, the different specificity profile revealed by our binding study suggests that the specific partner recognition by each WW domain might be an important factor for YAP functional modulation.

These results also suggest that high level of intrinsic specificity can be encoded within relatively short peptide sequences for these domains. Analysis of the structural models of the complexes, together with the phage display study of the binding preferences of these domains, indicate that electrostatic interactions play a very relevant role in determining binding specificity within the YAP WW domains. The significance of electrostatics in protein folding, binding and function is well established. In fact, it has been reported that the electrostatic potential is a distinguishing feature of WW domains, according to which they can be organized in four different categories [66]. In this sense, the two WW domains of YAP can clearly be classified in two different groups regarding to the degree of polarization of the binding site region. In the case of the PTCH1-b sequence, the high level of specificity has been achieved, not only by maximizing favorable interactions with the target, but also making unfavorable the interactions with off-target molecules, fine-tuning the electrostatic potential of the ligand. This observation is of interest, not only for the understanding of YAP function, but also from the design point of view, revealing the possibility of designing small ligands showing high specificity for one of these domains, of interest to delineate the functional role of each WW domain in YAP signaling and, eventually, of potential therapeutic value.

## Supporting Information

S1 Fig Stability curves for the individual WW domains of YAP. Gibbs energy changes for the thermal unfolding of YAP-WW1 (left panel) and YAP-WW2 (right panel) isolated domains resulting from the global fit of the DSC curves at several pH values. Solid lines with symbols represent the temperature dependence of the Gibbs energy function ΔG_N-U_(T) as a function of the pH: circles for pH 7.0, squares for pH 5.0, triangles for pH 4.0 and rhombi for pH 3.0. The arrows indicate the pH dependence of the T_S_ values.
(TIF)

S2 Fig Binding energetics of peptide ligands to YAP-WW1 (black bars) and YAP-WW2 (white bars). **A)** Dissociation constants for the LATS1-a, LATS1-b, LATS2, PTCH1-a and PTCH1-b peptide ligands for their interaction with the isolated WW domains of YAP. **B)** Enthalpic and entropic contributions to the binding affinity.
(TIF)

S3 Fig Induction of YAP Expression Results in Reduced Cell Attachment, which is Rescued by PTCH1 (top panel). HisMax-YAP or control vector were transfected into HEK293 cells that express Flag-PTCH1 WT in an inducible system. 24 hrs post transfection, the cells were distributed into new plates and the expression of Flag-PTCH1 WT was induced by tetracycline. O hr or 96 hrs post induction, cells were trypsinized and their numbers were counted. The growth rates in this 96 hrs are shown in the graph. The expression of induced Flag-PTCH1 and transfected YAP was monitored by immunoblotting. **PTCH1 impairs the ability of YAP to stabilize p73 (lower panel).** HEK293 cells that express Flag-PTCH1 WT or Flag-PTCH1 PY1*&2* mutant in an inducible system were transfected with HA-p73 and HisMax-YAP WT. 24hrs later, the cells were plated in fresh DMEM containing 1% FBS. Tetracycline was added to the medium to induce the expression of Flag-PTCH1 WT or mutant. 96hrs after induction, the cells were harvested, followed by immunoblotting using indicated antibodies.
(TIF)

S4 Fig Modelled structures of YAP-WW1 (left panels) and YAP-WW2 (right panels) in complex with A) PTCH1-a, B) PTCH1-b, C) LATS1-a, D) LATS1-b and E) LATS2 peptide ligands. YAP-WW1 and YAP–WW2 domains are shown as green and blue surfaces respectively. Peptide ligands and protein residues defining the canonical xP and xY pockets at the binding sites are shown as sticks. Non-conserved residues at the binding site are labelled in red. Hydrogen bonds interactions are shown as discontinuous black lines.
(TIF)

S1 Table Nature and identity of ionisable groups and contribution to the heat capacity (Fp·ΔC_p,prot_) of YAP WW domains. (DOC)

S2 Table Thermal denaturation parameters for the isolated YAP WW domains at different pH values. (DOC)

S3 Table Hydrogen-bonding interactions in modeled complexes of YAP WW domains. (DOCX)
