# Supplementary figures and images for: WW Domains of the Yes-Kinase-Associated-Protein (YAP) Transcriptional Regulator Behave as Independent Units with Different Binding Preferences for PPxY Motif-Containing Ligands
Source: PLoS One. 2015 Jan 21;10(1):e0113828. doi: 10.1371/journal.pone.0113828 (PMC4301871; doi:10.1371/journal.pone.0113828)

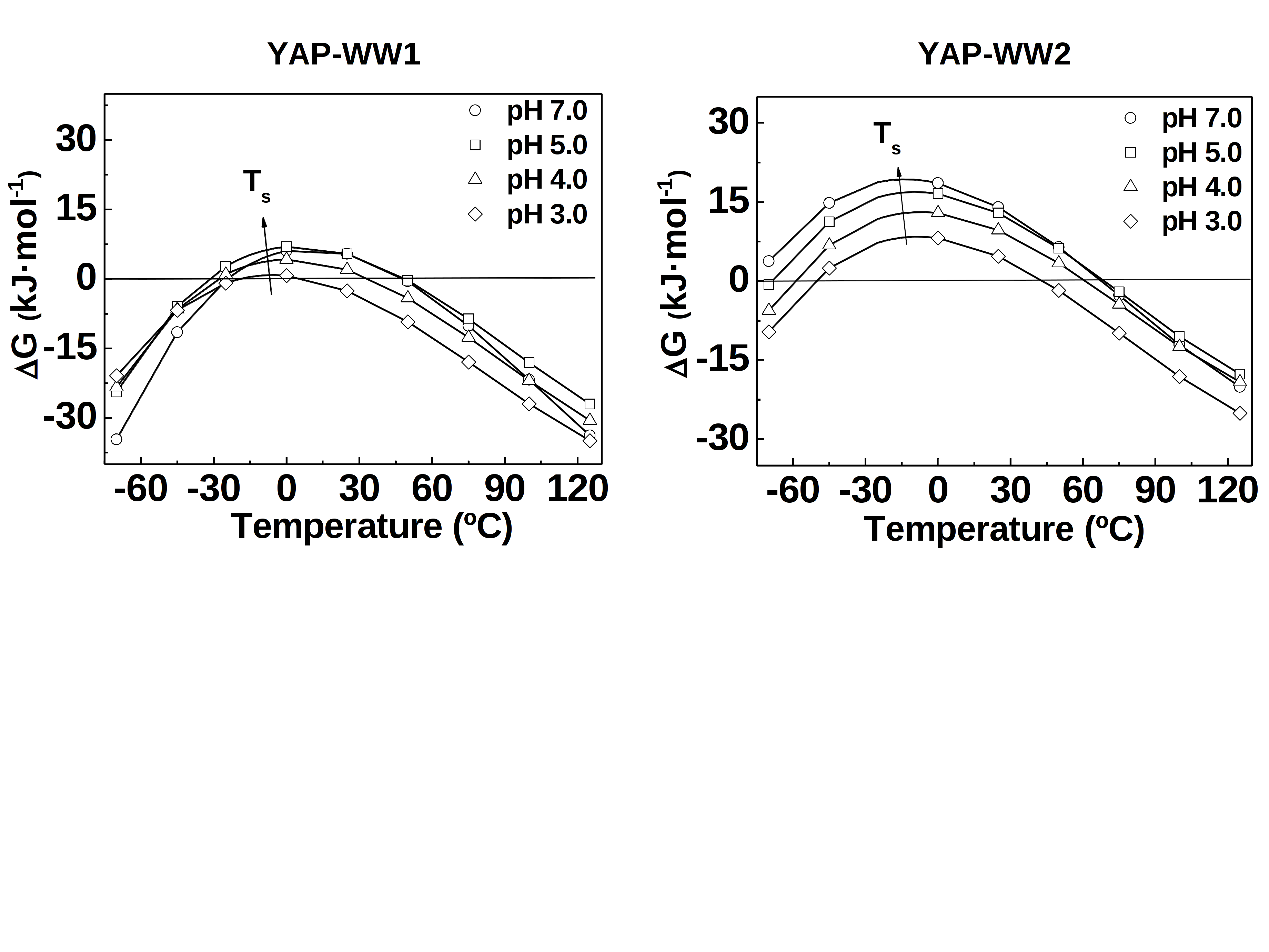

Supplement: S1 Fig — Gibbs energy changes for the thermal unfolding of YAP-WW1 (left panel) and YAP-WW2 (right panel) isolated domains resulting from the global fit of the DSC curves at several pH values. Solid lines with symbols represent the temperature dependence of the Gibbs energy function ΔGN-U(T) as a function of the pH: circles for pH 7.0, squares for pH 5.0, triangles for pH 4.0 and rhombi for pH 3.0. The arrows indicate the pH dependence of the TS values. (TIF) [file pone.0113828.s001.tif]

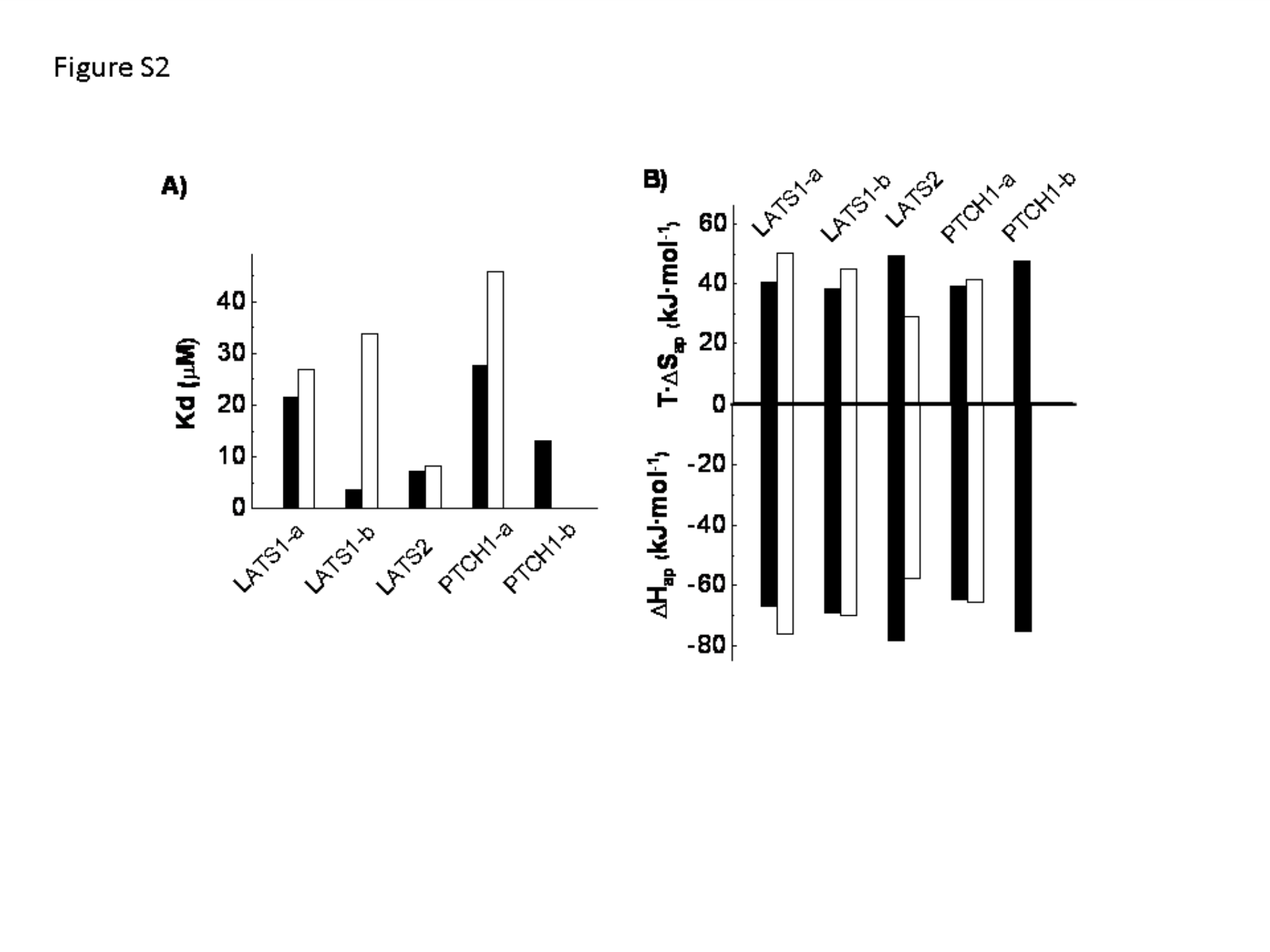

Supplement: S2 Fig — A) Dissociation constants for the LATS1-a, LATS1-b, LATS2, PTCH1-a and PTCH1-b peptide ligands for their interaction with the isolated WW domains of YAP. B) Enthalpic and entropic contributions to the binding affinity. (TIF) [file pone.0113828.s002.tif]

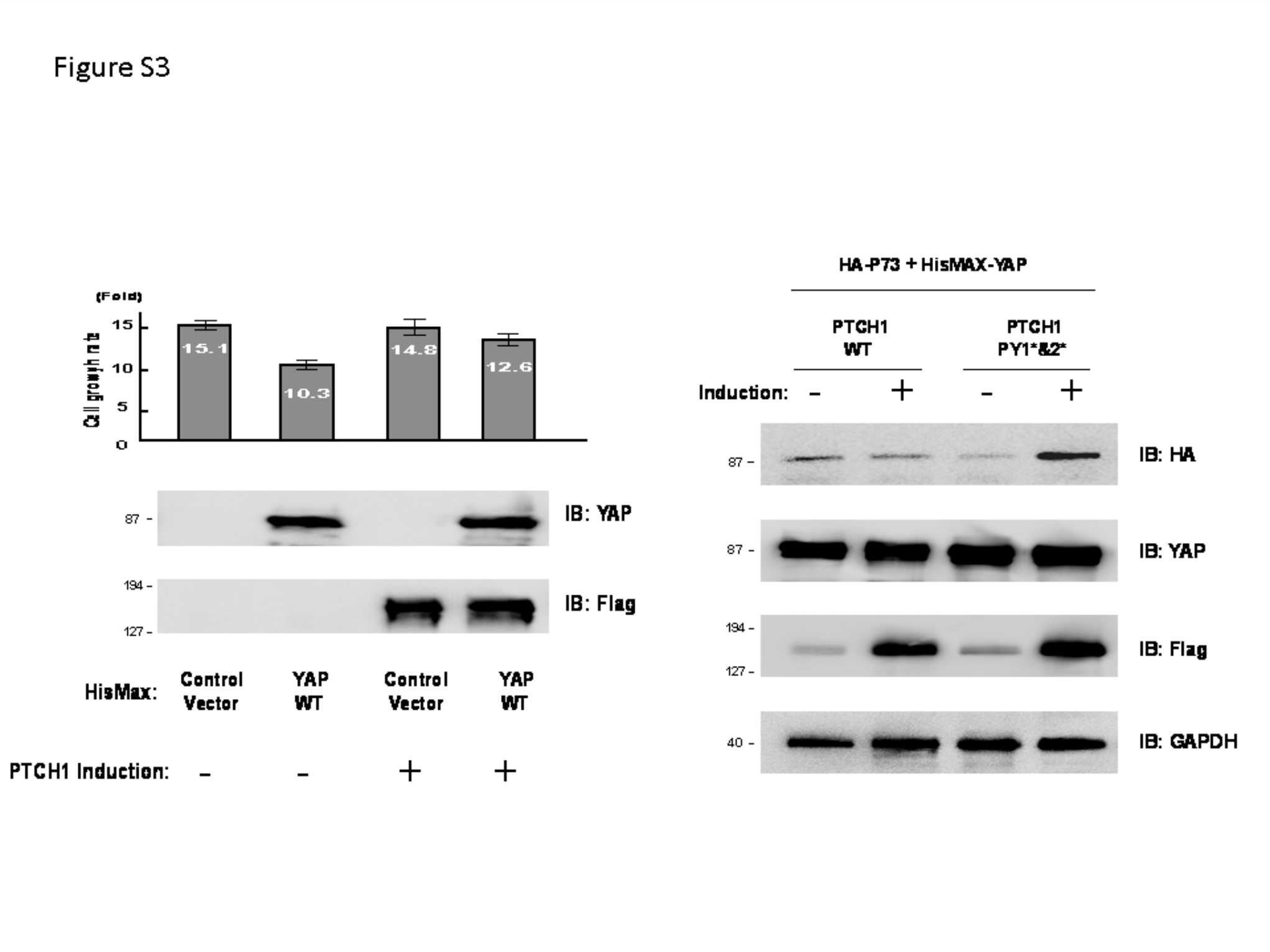

Supplement: S3 Fig — HisMax-YAP or control vector were transfected into HEK293 cells that express Flag-PTCH1 WT in an inducible system. 24 hrs post transfection, the cells were distributed into new plates and the expression of Flag-PTCH1 WT was induced by tetracycline. O hr or 96 hrs post induction, cells were trypsinized and their numbers were counted. The growth rates in this 96 hrs are shown in the graph. The expression of induced Flag-PTCH1 and transfected YAP was monitored by immunoblotting. PTCH1 impairs the ability of YAP to stabilize p73 (lower panel). HEK293 cells that express Flag-PTCH1 WT or Flag-PTCH1 PY1*&2* mutant in an inducible system were transfected with HA-p73 and HisMax-YAP WT. 24hrs later, the cells were plated in fresh DMEM containing 1% FBS. Tetracycline was added to the medium to induce the expression of Flag-PTCH1 WT or mutant. 96hrs after induction, the cells were harvested, followed by immunoblotting using indicated antibodies. (TIF) [file pone.0113828.s003.tif]

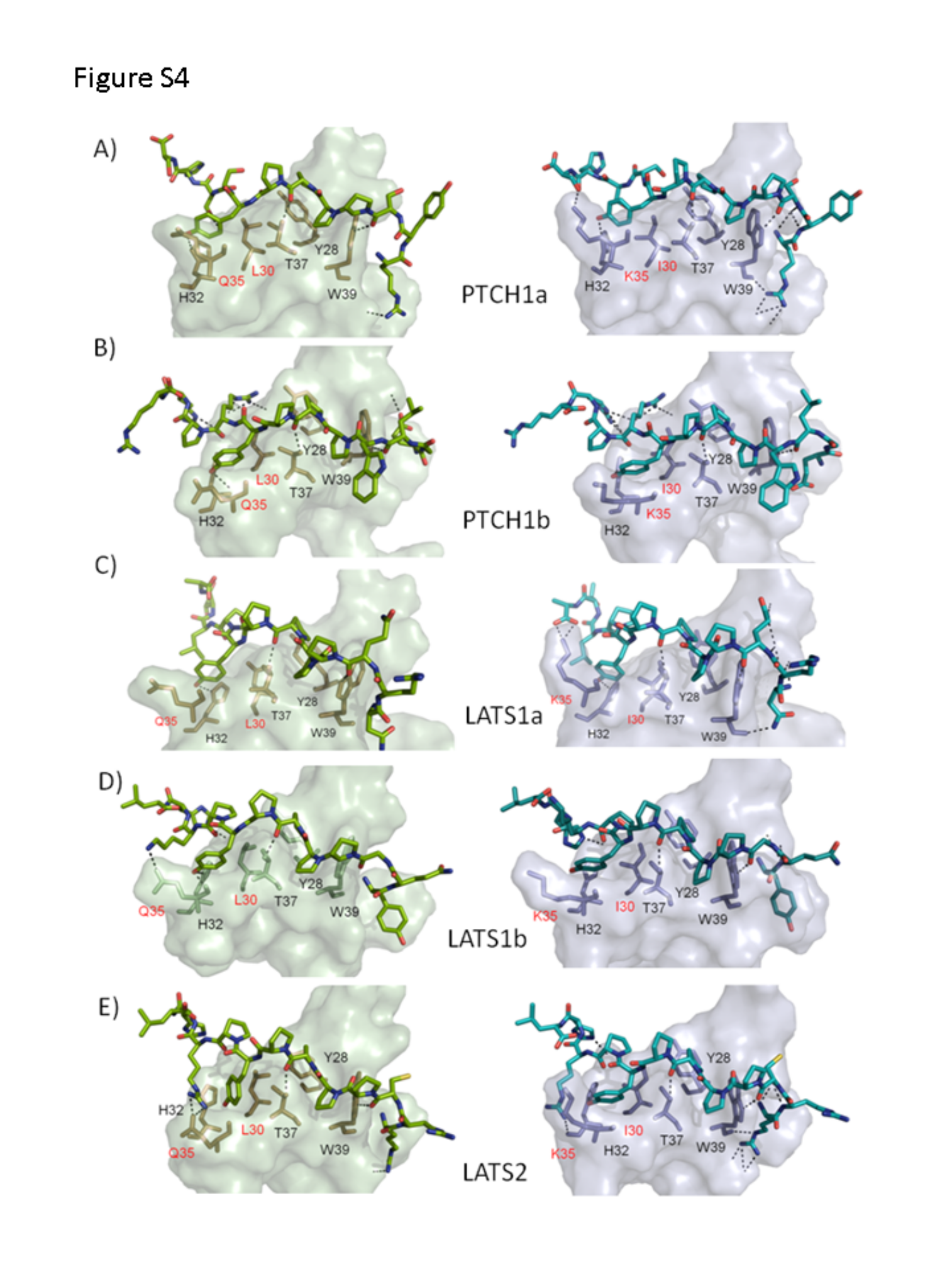

Supplement: S4 Fig — YAP-WW1 and YAP–WW2 domains are shown as green and blue surfaces respectively. Peptide ligands and protein residues defining the canonical xP and xY pockets at the binding sites are shown as sticks. Non-conserved residues at the binding site are labelled in red. Hydrogen bonds interactions are shown as discontinuous black lines. (TIF) [file pone.0113828.s004.tif]
