# Supplementary material for: WW Domains of the Yes-Kinase-Associated-Protein (YAP) Transcriptional Regulator Behave as Independent Units with Different Binding Preferences for PPxY Motif-Containing Ligands
Source: PLoS One. 2015 Jan 21;10(1):e0113828. doi: 10.1371/journal.pone.0113828 (PMC4301871; doi:10.1371/journal.pone.0113828)
Supplement: S1 Table — (DOC) [file pone.0113828.s005.doc]

| **WW domain** | **Ionisable group** | | **F_p_·ΔC_p,prot_,**  F_p,todos_=1  (J·mol^-1^·K^-1^) | ***F_p_·ΔC_p,prot,max_ ,** F_p,His_=0.5  (J·mol^-1^·K^-1^) |
| --- | --- | --- | --- | --- |
| **YAP-WW1** | **His** | 1 | 16.7 | 304.7 |
|  | **Glu** | 2 | 250.8 | 250.8 |
|  | **Asp** | 4 | 501.6 | 501.6 |
|  | **C-terminal** | 1 | 125.4 | 125.4 |
|  | **Total** |  | 894.5 | 1182.5 |
| **YAP-WW2** | **His** | 1 | 16.7 | 304.7 |
|  | **Glu** | 2 | 250.8 | 250.8 |
|  | **Asp** | 4 | 376.2 | 376.2 |
|  | **C-terminal** | 1 | 125.4 | 125.4 |
|  | **Total** |  | 894.5 | 1182.5 |
| Enthalpy values for the difference between ionisation states for carboxylic and indole groups are 125.4 J mol^-1^·K^-1^ and -2 J·mol^-1^, respectively. Heat capacity values for the difference between ionisation states for carboxylic and indole groups are 16.7 J mol^-1^ K^-1^ and 29.6 J mol^-1^, respectively [2]  *To calculate the maximum heap capacity values (ΔC_p,prot,max_), we consider F_p_ = 1 for all ionisable groups except for histidine that it is F_p_ = 0.5, assuming that the pH coincides with their pK_a_ (when thermal fluctuation is maximum). The thermal fluctuation was calculated using the next equation$F_{p}\left(1-F_{p}\right)\Delta H_{p}^{2}/RT^{2}$ [2]. | | | | |
