# Supplementary material for: WW Domains of the Yes-Kinase-Associated-Protein (YAP) Transcriptional Regulator Behave as Independent Units with Different Binding Preferences for PPxY Motif-Containing Ligands
Source: PLoS One. 2015 Jan 21;10(1):e0113828. doi: 10.1371/journal.pone.0113828 (PMC4301871; doi:10.1371/journal.pone.0113828)
Supplement: S2 Table — (DOC) [file pone.0113828.s006.doc]

|  | **YAP-WW1** | | | **YAP-WW2** | | |
| --- | --- | --- | --- | --- | --- | --- |
| **pH** | **ΔH_m,exp_**  **(kJ·mol^-1^)** | **T_m_**  **(ºC)** | **F_p_** | **ΔH_m,exp_**  **(kJ·mol^-1^)** | **T_m_**  **(ºC)** | **F_p_** |
| **7.0** | 101 | 48.5 | 1.0 | 128 | 67.8 | 0.85 |
| **5.0** | 93 | 49.1 | 0.5 | 117 | 69.0 | 0.5 |
| **4.0** | 72 | 34.7 | 0.3 | 105 | 61.3 | 0.5 |
| **3.0** | 32 | 9.4 | 0.1 | 90 | 43.9 | 0.0 |
|  | **ΔC_p,p_^b^ =** 1.0 kJ·mol^-1^·K^-1^  **T_S_** = -2 ºC | | | **ΔC_p,p_^b^ =** 1.0 kJ·mol^-1^·K^-1^  **T_S_** = -15 ºC | | |
| **Baselines^c^** | | | | | | |
| **C_p,N_(T)** | 9.7 - 0.044·(T-T_r_) | | | 9.6 - 0.044·(T-T_r_) | | |
| **C_p,D_(T)** | 11.4 +0.023·(T-T_r_)–10.2·10^-5^·(T-T_r_)^2^ | | | 11.2 +0.024·(T-T_r_)–10.3·10^-5^·(T-T_r_)^2^ | | |
| ^a^ Errors have been estimated to be 5% (in degree Celsius), 10% for ΔH_m_, and 20% for the rest of the parameters. ^b^ Protonation heat capacity changes, ΔC_p,p_, were obtained from the difference between the C_p_ value at 100 °C in the DSC curves at pH 3.0 and at pH 7.0. ^c^ Baselines resulting from the fitting, where T_r_ is the reference temperature and the underlined values were estimated from the individual contributions of amino acids and chemical groups [4]. | | | | | | |
