# Supplementary material for: WW Domains of the Yes-Kinase-Associated-Protein (YAP) Transcriptional Regulator Behave as Independent Units with Different Binding Preferences for PPxY Motif-Containing Ligands
Source: PLoS One. 2015 Jan 21;10(1):e0113828. doi: 10.1371/journal.pone.0113828 (PMC4301871; doi:10.1371/journal.pone.0113828)
Supplement: S3 Table — (DOCX) [file pone.0113828.s007.docx]

**Table S3. Hydrogen-bonding interactions in modeled complexes of YAP WW domains.**

| **YAP-WW1** | | | | | | | | **YAP-WW2** | | | | | | | |
| --- | --- | --- | --- | --- | --- | --- | --- | --- | --- | --- | --- | --- | --- | --- | --- |
|  | **Donor** | | | **Aceptor** | | |  | **Donor** | | | **Aceptor** | | |  |  |
|  | **Res** | **Atom** | **SS^1^** | **Res** | **Atom** | **SS^1^** | **Dist ^2^(Å)** | **Res** | **Atom** | **SS^1^** | **Res** | **Atom** | **SS^1^** | **Dist^2^ (Å)** |  |
| **PTCH1a** | W39 | Nε | C-ter | S574 | CO | Ligand | 2.2 | W39 | Nε | C-ter | S574 | CO | Ligand | 1.9 | * |
|  | T37 | Oγ | Strβ3 | P577 | CO | Ligand | 1.7 | T37 | Oγ | Strβ3 | P577 | CO | Ligand | 1.9 | * |
|  | Y579 | Oε | Ligand | H32 | Nδ1 | Lpβ2-β3 | 1.9 | Y579 | Oε | Ligand | H32 | Nδ1 | Lpβ2-β3 | 1.9 | * |
|  | S581 | Oε | Ligand | E18 | Oε1 | Strβ1 | 2.2 | S583 | Oε | Ligand | E18 | Oε1 | Strβ1 | 2.0 |  |
|  |  |  |  |  |  |  |  | R572  Y573  S574 | NH  NH  NH | Ligand  Ligand  Ligand | E26  E26  E26 | Oε2  Oε2  Oε2 | Strβ2  Strβ2  Strβ2 | 2.0  1.9  2.5 |  |
|  | R572 | Nη1 | Ligand | Q40 | Oε | C-ter | 1.8 | R572 | Nη2 | Ligand | D45 | Oδ1 | C-ter | 1.7 |  |
|  |  |  |  |  |  |  |  | R572 | Nη1 | Ligand | D45 | Oδ2 | C-ter | 1.8 |  |
| **PTCH1b** | W39 | Nε | C-ter | L1245 | CO | Ligand | 1.8 | W39 | Nε | C-ter | L1245 | CO | Ligand | 2.0 | * |
|  | T37 | Oγ | Strβ3 | P1248 | CO | Ligand | 1.8 | T37 | Oγ | Strβ3 | P1248 | CO | Ligand | 1.9 | * |
|  | Y1250 | Oε | Ligand | H32 | Nδ1 | Lpβ2-β3 | 2.4 | Y1250 | Oε | Ligand | H32 | Nδ1 | Lpβ2-β3 | 2.2 | * |
|  | R1251 | Nη1 | Ligand | E18 | Oε2 | Strβ1 | 1.9 | R1251 | Nη1 | Ligand | E18 | Oε2 | Strβ1 | 1.9 | * |
|  | R1251 | Nη2 | Ligand | E18 | Oε2 | Strβ1 | 1.8 | R1251 | Nη2 | Ligand | E18 | Oε2 | Strβ1 | 1.8 | * |
|  | R1253 | Nη1 | Ligand | E18 | Oε1 | Strβ1 | 1.7 | R1253 | Nη1 | Ligand | E18 | Oε1 | Strβ1 | 2.4 | * |
|  | R1253 | Nη2 | Ligand | E18 | Oε1 | Strβ1 | 2.2 | R1253 | Nη2 | Ligand | E18 | Oε1 | Strβ1 | 1.7 | * |
|  | R1251 | Nη2 | Ligand | M19 | CO | Strβ1 | 2.1 | R1251 | Nη2 | Ligand | Q19 | CO | Strβ1 | 2.2 |  |
|  | Y1250 | Oε | Ligand | N31 | CO | Strβ2 | 2.3 |  |  |  |  |  |  |  |  |
|  | Q26 | Nε2 | Strβ2 | E1243 | Oε2 | Ligand | 1.8 |  |  |  |  |  |  |  |  |
| **LATS1a** | W39 | Nε | C-ter | Q371 | CO | Ligand | 2.0 | W39 | Nε | C-ter | Q371 | CO | Ligand | 1.8 | * |
|  | T37 | Oγ | Strβ3 | P374 | CO | Ligand | 1.7 | T37 | Oγ | Strβ3 | P374 | CO | Ligand | 2.0 | * |
|  | Y376 | Oε | Ligand | H32 | Nδ1 | Lpβ2-β3 | 2.1 | Y376 | Oε | Ligand | H32 | Nδ1 | Lpβ2-β3 | 1.9 | * |
|  | N369 | Nδ2 | Ligand | Q40 | Oε1 | C-ter | 1.9 | N369  Q371  Q371 | NH  NH  Nε2 | Ligand  Ligand  Ligand | E26  E26  E26 | Oε2  Oε2  Oε2 | Strβ2  Strβ2  Strβ2 | 1.9  2.0  1.9 |  |
|  |  |  |  |  |  |  |  | R370 | NH | Ligand | E26 | Oε1 | Strβ2 | 1.8 |  |
|  |  |  |  |  |  |  |  | Q371 | Nε2 | Ligand | D24 | Oδ1 | Lpβ1-β2 | 1.8 |  |
| **LATS1b** | W39 | Nε | C-ter | G554 | CO | Ligand | 2.0 | W39 | Nε | C-ter | G554 | CO | Ligand | 1.9 | * |
|  | T37 | Oγ | Strβ3 | P557 | CO | Ligand | 1.7 | T37 | Oγ | Strβ3 | P557 | CO | Ligand | 1.8 | * |
|  | Y559 | Oε | Ligand | H32 | Nδ1 | Lpβ2-β3 | 2.1 | Y559 | Oε | Ligand | H32 | Nδ1 | Lpβ2-β3 | 2.0 | * |
|  | H562 | Nε2 | Ligand | E18 | Oε2 | Strβ1 | 1.9 | H562 | Nε2 | Ligand | E18 | Oε2 | Strβ1 | 1.8 | * |
|  | K561 | Nζ | Ligand | Q35 | Oε | Strβ3 | 1.8 | Y552  Q553 | NH  NH | Ligand  Ligand | E26  E26 | Oε2  Oε1 | Strβ2  Strβ2 | 2.0  2.1 |  |
| **LATS2** | W39 | Nε | C-ter | C513 | CO | Ligand | 1.9 | W39 | Nε | C-ter | C513 | CO | Ligand | 2.0 | * |
|  | T37 | Oγ | Strβ3 | P516 | CO | Ligand | 1.8 | T37 | Oγ | Strβ3 | P516 | CO | Ligand | 1.8 | * |
|  | K520 | Nζ | Ligand | H32 | Nδ1 | Lpβ2-β3 | 2.3 | Y518 | Oε | Ligand | H32 | Nδ1 | Lpβ2-β3 | 2.1 |  |
|  | R511 | Nη2 | Ligand | E6 | Oε2 | N-ter | 1.7 | R511 | Nη1 | Ligand | D45 | Oδ2 | C-ter | 1.8 |  |
|  | R511 | Nη2 | Ligand | Q40 | Oε1 | C-ter | 2.1 | R511 | Nη2 | Ligand | D45 | Oδ1 | C-ter | 1.7 |  |
|  |  |  |  |  |  |  |  | R511 | Nε | Ligand | W39 | CO | C-ter | 2.3 |  |
|  |  |  |  |  |  |  |  | R511  R512  C513 | NH  NH  NH | Ligand  Ligand  Ligand | E26  E26  E26 | Oε2  Oε2  Oε2 | Strβ2  Strβ2  Strβ2 | 1.8  1.8  1.8 |  |
|  |  |  |  |  |  |  |  | H521 | Nε2 | Ligand | E18 | Oε2 | Strβ1 | 1.9 |  |
| ^1^ SS are the secondary structure element where each residue (Res) is found. Lp and Str are abbreviations for loop and strand, respectively.  ^2^ Reported are distances between hydrogen and acceptor atoms.  * Hydrogen bonds common to WW1 and WW2 complexes. | | | | | | | | | | | | | | | |
